# Association between systemic inflammation response index and atrial fibrillation in chronic obstructive pulmonary disease: a multicenter cross-sectional study

**DOI:** 10.3389/fmed.2025.1666658

**Published:** 2025-09-03

**Authors:** Hao Xu, Tianye Li, Mengya Yang, Yanhong Zheng, Xiaolong Zhu, Lijuan Chen, Hongjun Zhao

**Affiliations:** ^1^Zhejiang Province Engineering Research Center for Endoscope Instruments and Technology Development, Clinical Research Center, Department of Pulmonary and Critical Care Medicine, Quzhou People's Hospital, The Quzhou Affiliated Hospital of Wenzhou Medical University, Quzhou, China; ^2^Department of Pulmonary and Critical Care Medicine, Key Laboratory of Interventional Pulmonology of Zhejiang Province, The First Affiliated Hospital of Wenzhou Medical University, Wenzhou, China; ^3^Gansu Provincial Centre for Disease Control Land Prevention, Lanzhou, China

**Keywords:** systemic inflammation response index, chronic obstructive pulmonary disease, atrial fibrillation, risk prediction, cross-sectional study

## Abstract

**Background:**

Systemic inflammation plays a critical role in the pathogenesis of both chronic obstructive pulmonary disease (COPD) and atrial fibrillation (AF). As an emerging biomarker reflecting systemic inflammatory status, the association between the systemic inflammation response index (SIRI) and the risk of AF in patients with COPD remains unclear. Therefore, this study aimed to investigate the association between SIRI levels and the risk of AF in patients with COPD.

**Methods:**

In this multicenter cross-sectional study, we enrolled 1,133 hospitalized COPD patients from May 2021 to May 2024 at the First Affiliated Hospital of Wenzhou Medical University and Quzhou People's Hospital. Patients were categorized into four groups based on SIRI quartiles. We used multivariate logistic regression, restricted cubic spline (RCS) analysis, and receiver operating characteristic (ROC) curves to evaluate the association between SIRI and AF risk. Subgroup, interaction, and sensitivity analyses were conducted to assess robustness and effect modification.

**Results:**

Among the study population, 225 patients (19.85%) had AF. After full adjustment, this cross-sectional analysis demonstrates that each 1-unit increase in SIRI was associated with a 4.6% higher AF risk (OR = 1.046, 95% CI: 1.018–1.075, *p* = 0.002). Patients in the highest SIRI quartile had a 116.2% increased risk compared to those in the lowest quartile (OR = 2.162, 95% CI: 1.325–3.527, *p* = 0.002). RCS analysis revealed a significant linear dose–response relationship between SIRI and AF risk. ROC analysis showed that combining SIRI with conventional risk factors improved predictive accuracy for AF (AUC = 0.818, 95% CI: 0.787–0.848, *p* < 0.001). A significant interaction was observed among smokers (interaction *p* = 0.006), and results remained robust in sensitivity analyses.

**Conclusion:**

Elevated SIRI levels are independently associated with an increased risk of AF in patients with COPD, especially among smokers. As a simple and readily accessible biomarker of low-grade systemic inflammation, SIRI may serve as an effective tool for assessing the risk of atrial fibrillation in patients with COPD.

## 1 Introduction

Chronic obstructive pulmonary disease (COPD) is a systemic inflammatory disease characterized by persistent airflow limitation and chronic airway inflammation. Clinically, it manifests with progressively worsening symptoms, such as dyspnea, chronic cough, and sputum production, and is often associated with significant systemic effects (1). Increasing evidence indicates that the systemic inflammatory response in COPD not only exacerbates pulmonary damage but also plays a critical role in the development of extrapulmonary comorbidities, including cardiovascular diseases (2–4). Among these, atrial fibrillation (AF) is one of the most common arrhythmias in COPD patients and has been closely linked to poor clinical outcomes and increased mortality (5, 6). Desai et al. reported that ~30% of hospitalized patients with COPD developed cardiac arrhythmias, with AF accounting for 22.1% of these cases. Moreover, the incidence of new-onset AF in COPD patients was found to be nearly twice that of individuals without COPD (7). In addition, a large retrospective study from the United States revealed that the prevalence of AF among COPD patients increased from 12.9% in 2003 to 21.3% in 2014 (8). The latest analysis from the GLORIA-AF registry also showed that COPD patients with AF have worse prognoses, including higher mortality rates, major adverse cardiovascular events (MACE), and bleeding complications (6). Inflammation-induced atrial remodeling, oxidative stress, and autonomic dysfunction are considered important underlying mechanisms linking COPD to AF (9–11). COPD patients often exhibit elevated levels of various pro-inflammatory factors, such as interleukin-6 (IL-6), C-reactive protein (CRP), and tumor necrosis factor-alpha (TNF-α), which contribute to atrial structural remodeling and electrophysiological abnormalities, key factors in the onset of AF (10, 12, 13). Despite the close relationship between COPD and AF, there is a lack of reliable, easily detectable biomarkers that can simultaneously reflect the systemic inflammatory status and predict AF risk in COPD patients. Therefore, the exploration of novel inflammation-related biomarkers is of significant clinical importance for the early identification of high-risk patients.

The systemic inflammation response index (SIRI) is a novel inflammatory marker calculated based on the peripheral blood counts of neutrophils, monocytes, and lymphocytes, and has been widely used in recent years to assess low-grade chronic inflammation. Compared to traditional inflammatory markers such as C-reactive protein (CRP) or the neutrophil-to-lymphocyte ratio (NLR), SIRI provides a more comprehensive reflection of the interaction between inflammation and the immune system, offering higher sensitivity and stability in the context of chronic systemic inflammation (14). Recent studies have demonstrated that the SIRI exhibits excellent predictive value for risk stratification and prognosis in various diseases, including coronary heart disease, stroke, and diabetes. For example, a retrospective cohort study by Lin et al. (15) identified SIRI as a potential biomarker for atrial fibrillation risk in patients with ischemic stroke. Similarly, a large cross-sectional study by Wang et al. (16) found that elevated SIRI levels were significantly associated with metabolic dysfunction–associated fatty liver disease (MAFLD). In addition, Ma et al. (17) reported that, among patients with ischemic heart failure (IHF) undergoing percutaneous coronary intervention, an increased SIRI was an independent risk factor for major adverse cardiovascular events (MACE). However, to date, no large-scale studies have systematically assessed the relationship between SIRI and AF risk in COPD patients.

Given the chronic inflammatory state of COPD and the inflammation-related mechanisms underlying atrial fibrillation (AF), we hypothesized that SIRI may serve as a potential biomarker for AF risk stratification in this population. Therefore, the aim of this study was to investigate the association between SIRI levels and the occurrence of AF in COPD patients and to assess its potential application in clinical risk assessment. The findings of this study may provide new insights into the pathophysiological mechanisms and risk stratification strategies for COPD patients with AF.

## 2 Methods

### 2.1 Study population

**Figure 1:**
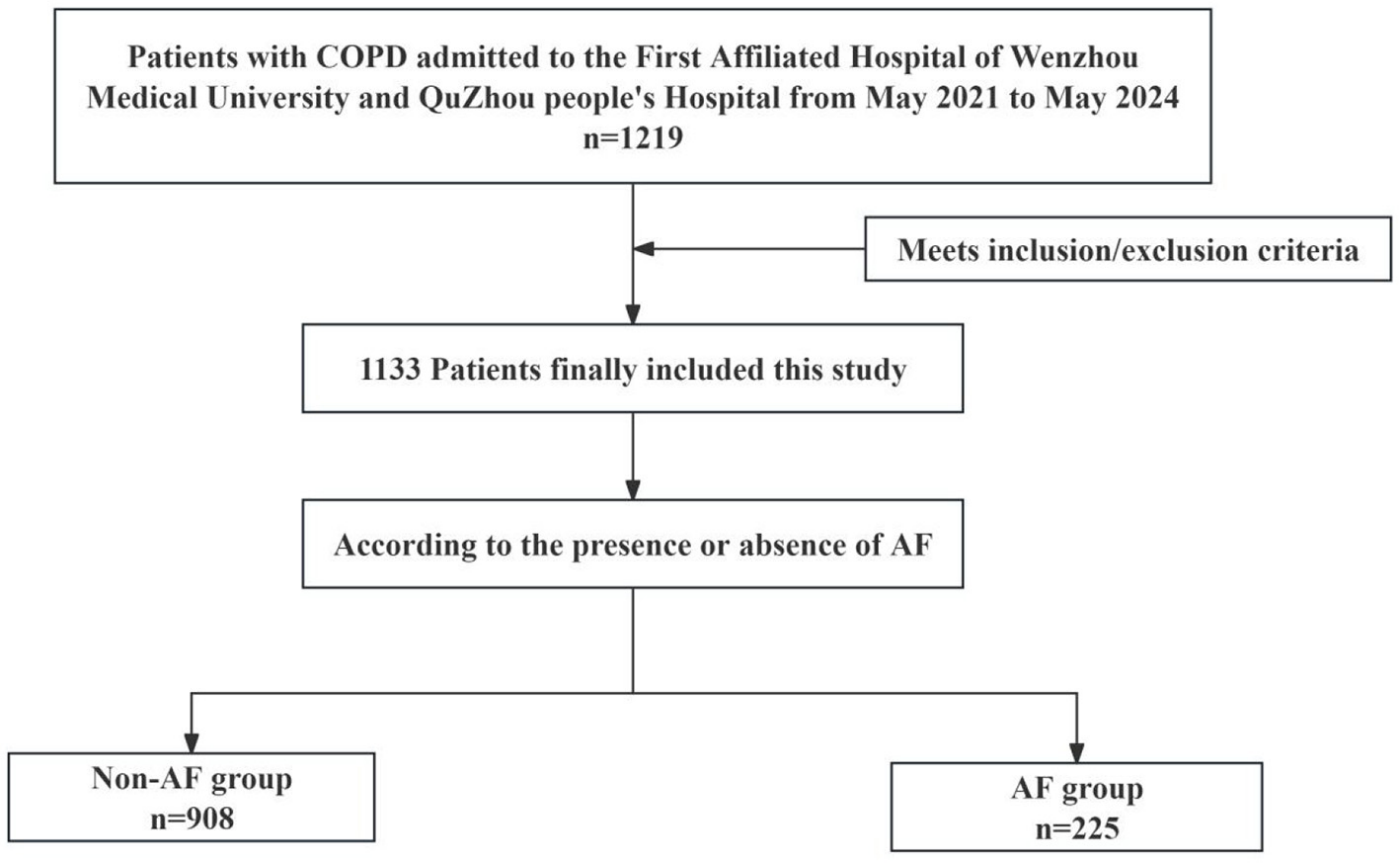
The study's flow diagram. AF, atrial fibrillation.

This multicenter, cross-sectional study included patients with COPD who were hospitalized between May 2021 and May 2024 at the First Affiliated Hospital of Wenzhou Medical University and Quzhou People's Hospital. All clinical data were retrospectively collected from structured fields within the institutional electronic medical record (EMR) systems. Inclusion criteria were as follows: (1) COPD was diagnosed in accordance with the 2023 Global Initiative for Chronic Obstructive Lung Disease (GOLD) guidelines (1); (2) age ≥ 18 years; (3) availability of complete medical history and laboratory/examination data. Exclusion criteria were as follows: (1) significant missing baseline data; (2) presence of psychiatric disorders or cognitive impairment; (3) severe comorbidities, including advanced hepatic or renal failure, or terminal malignancies; (4) patients with diseases related to atrial fibrillation, such as structural cardiomyopathies (dilated, hypertrophic, or restrictive), valvular heart disease (rheumatic or degenerative), atrial septal defects (e.g., congenital atrial septal defect), or hyperthyroidism; (5) pregnant or lactating women. A total of 1,133 patients were ultimately included in the study (Figure 1). The study protocol was conducted in accordance with the Declaration of Helsinki and was approved by the Ethics Committees of the First Affiliated Hospital of Wenzhou Medical University and Quzhou People's Hospital (Approval No. KY2025-R206 and 2025-076).

### 2.2 The calculation and grouping of SIRI

Fasting venous blood samples were collected by trained nursing staff upon patient admission. Neutrophil, lymphocyte, and monocyte counts were measured in the hospital's certified clinical laboratory using standard procedures. The SIRI was calculated using the following formula: SIRI = (monocyte count × neutrophil count)/lymphocyte count. Patients were stratified into quartiles based on SIRI values: Q1 ( ≤ 1.22, *n* = 282), Q2 (1.22–2.08, *n* = 284), Q3 (2.08–3.98, *n* = 283), and Q4 (>3.98, *n* = 284). In addition, SIRI was standardized using the formula: Standardized SIRI = (raw SIRI—mean SIRI)/standard deviation of SIRI.

### 2.3 Definition and classification of atrial fibrillation

According to the 2024 guidelines of the European Society of Cardiology (ESC), confirmation by an electrocardiogram (ECG; 12-lead, multi-lead, or single-lead) is recommended to establish the diagnosis of clinical AF (18). Specifically, a standard 12-lead ECG or a multi-lead or single-lead ECG recording of ≥30 s showing heart rhythm with no discernible repeating P waves and irregular RR intervals (when atrioventricular conduction is not impaired) is diagnostic of clinical AF (19). All patients were classified into the AF group and non-AF group based on current or previous ECG results.

### 2.4 Collection and definition of covariates

All data were extracted from the hospital's electronic medical record (EMR), including demographic information, comorbidities, auxiliary examination, and blood biomarkers. Demographic variables included age, gender (male or female), smoking (yes or no), and drinking (yes or no). Smoking was defined as regular smoking in the past, regardless of current abstinence. Drinking defined as a history of regular alcohol consumption, regardless of whether the patient currently abstains. The comorbidities included asthma, heart failure, hypertension, diabetes mellitus, cerebrovascular disease, coronary artery disease, a personal history of malignant tumors, and pulmonary arterial hypertension. Asthma was defined as a previously confirmed diagnosis, current treatment with anti-asthma medications, or the presence of typical respiratory symptoms with reversible airflow limitation (defined as an increase in FEV_1_ of ≥12% and ≥200 ml after bronchodilator administration) (20). Heart failure was defined as ongoing treatment with heart failure medications or typical signs and symptoms of heart failure accompanied by a left ventricular ejection fraction (LVEF) < 50% or a B-type natriuretic peptide (BNP) level ≥100 pg/ml (21). Hypertension was defined as a prior diagnosis or a measured systolic/diastolic blood pressure (SBP/DBP) ≥140/90 mmHg during hospitalization (22). Diabetes mellitus was defined as a prior diagnosis, current antidiabetic treatment, a fasting blood glucose level ≥7.0 mmol/L, or glycated hemoglobin (HbA1c) >6.5% (23). Cerebrovascular disease was defined as a history of stroke or transient ischemic attack (TIA), or neuroimaging-confirmed cerebrovascular events (CT or MRI), including ischemic stroke, hemorrhagic stroke, or TIA (24, 25). Coronary heart disease was defined as a previously diagnosed or ≥50% stenosis detected by coronary angiography or coronary CT angiography (26). A personal history of malignant tumors was defined as a history of malignant tumors that had been previously diagnosed and treated (e.g., with surgery, chemotherapy, or radiotherapy), but excluded patients who were currently in the advanced or terminal stage of the disease (27). Auxiliary examination data included pulmonary arterial hypertension diagnosed by echocardiography and the measured left ventricular ejection fraction (LVEF). Biomarkers included anthropometric and blood-based indicators. Among the anthropometric indicators, body mass index (BMI) was recorded and calculated as weight in kilograms divided by the square of height in meters (kg/m^2^). Blood biomarkers included hemoglobin (Hb), red blood cell (RBC), white blood cell (WBC), platelet (PLT), neutrophil (N), lymphocyte (L), monocyte (M), alanine aminotransferase (ALT), aspartate aminotransferase (AST), albumin (ALB), blood urea nitrogen (BUN), creatinine (Cr), uric acid (UA), total cholesterol (TC), low-density lipoprotein cholesterol (LDL-C), high-density lipoprotein cholesterol (HDL-C), and random glucose.

### 2.5 Statistical analysis

Continuous variables were expressed as mean ± standard deviation (SD) or median (interquartile range), depending on the normality of distribution, and compared using the independent samples *t*-test or non-parametric tests. Categorical variables were presented as frequencies (percentages) and compared using the chi-square test or Fisher's exact test, as appropriate. Missing covariates were imputed using multiple imputation with the mice package in R. Statistical analyses were performed using R (version 4.4.2) and SPSS (version 25.0). All statistical tests were two-sided, and a *p*-value < 0.05 was considered statistically significant.

To investigate the association between the SIRI and the occurrence of AF, logistic regression models were constructed. Univariate logistic regression was first performed to evaluate the association between each variable and AF. Variables with a *p*-value < 0.05 in the univariate analysis were subsequently included in a multivariate logistic regression model to further assess the independent association between SIRI and AF. SIRI was included in the models both as a continuous variable and as a categorical variable based on quartiles. The *p* for trend was calculated by treating the median value of SIRI within each quartile as a continuous variable in the model. To better illustrate the relationship between SIRI and AF, we constructed a restricted cubic spline (RCS) model to explore potential non-linear associations. Receiver operating characteristic (ROC) curve analysis was performed to assess the ability of SIRI to predict the occurrence of AF. The area under the curve (AUC) was calculated, and the DeLong test was used to compare the discriminatory power of different logistic regression models. Subgroup and interaction analyses were conducted to further validate the association between SIRI and AF. The analyses were stratified by age (< 75 and ≥75 years), sex (male and female), body mass index (BMI: < 18.5, 18.5–23.9, and ≥24 kg/m^2^), smoking status (yes or no), drinking status (yes or no), heart failure (yes or no), hypertension (yes or no), diabetes (yes or no), and coronary artery disease (yes or no). Finally, to ensure the robustness of the study findings, two additional sensitivity analyses were performed. Firstly, patients were stratified based on alternative SIRI grouping methods, including tertiles, median, and mean values, to examine whether the association with AF remained consistent. Secondly, patients with asthma, cerebrovascular disease, malignancy, or pulmonary hypertension were excluded to evaluate the stability of the results in a more homogeneous population.

## 3 Results

### 3.1 Demographic and clinical characteristics of patients with and without atrial fibrillation

A total of 1,133 patients were included in this study, among whom 225 (19.85%) had AF. As shown in Table 1, patients in the AF group were significantly older, had a lower proportion of smokers, and exhibited a higher prevalence of heart failure, hypertension, cerebrovascular disease, coronary artery disease, and pulmonary hypertension (all *p* < 0.05). In contrast, the prevalence of asthma and malignancy was significantly lower (both *p* < 0.05). Additionally, BMI, AST, BUN, Cr, UA, SIRI levels and the proportion in the highest quartile (Q4) were significantly higher in the AF group, while Hb, RBC, PLT, L, ALB, TC, LDL-C, and LVEF were significantly lower (all *p* < 0.05).

**Table 1:** Baseline demographic and clinical characteristics of the study population, by AF status.

**Variables**	**Total population**	**Non-AF**	**AF**	***p*-value**
	***N*** = **1,133**	***N*** = **908**	***N*** = **225**	
Age, years	73 (67, 81)	72 (65, 79)	79 (73, 84)	< 0.001
**Sex**, ***n*** **(%)**				
Male	973 (85.88)	786 (86.56)	187 (83.11)	0.183
Female	160 (14.12)	122 (13.44)	38 (16.89)	
**Smoking**, ***n*** **(%)**				
Yes	667 (58.87)	548 (60.35)	119 (52.89)	0.042
No	466 (41.13)	360 (39.65)	106 (47.11)	
**Drinking**, ***n*** **(%)**				
Yes	407 (35.92)	328 (36.12)	79 (35.11)	0.777
No	726 (64.08)	580 (63.88)	146 (64.89)	
**Asthma**, ***n*** **(%)**				
Yes	31 (2.74)	30 (3.30)	1 (0.44)	0.019
No	1,102 (97.26)	878 (96.70)	224 (99.56)	
**Heart failure**, ***n*** **(%)**				
Yes	144 (12.71)	78 (8.59)	66 (29.33)	< 0.001
No	989 (87.29)	830 (91.41)	159 (70.67)	
**Hypertension**, ***n*** **(%)**				
Yes	453 (39.98)	344 (37.89)	109 (48.44)	0.004
No	680 (60.02)	564 (62.11)	116 (51.56)	
**Diabetes**, ***n*** **(%)**				
Yes	165 (14.56)	132 (14.54)	33 (14.67)	0.961
No	968 (85.44)	776 (85.46)	192 (85.33)	
**Cerebrovascular disease**, ***n*** **(%)**				
Yes	124 (10.94)	70 (7.71)	54 (24.00)	< 0.001
No	1,009 (89.06)	838 (92.29)	171 (76.00)	
**Coronary heart disease**, ***n*** **(%)**				
Yes	113 (9.97)	77 (8.48)	36 (16.00)	< 0.001
No	1,020 (90.03)	831 (91.52)	189 (84.00)	
**Malignant tumors**, ***n*** **(%)**				
Yes	258 (22.77)	219 (24.12)	39 (17.33)	0.030
No	875 (77.23)	689 (75.88)	186 (82.67)	
**Pulmonary hypertension**, ***n*** **(%)**				
Yes	484 (42.72)	361 (39.76)	123 (54.67)	< 0.001
No	649 (57.28)	547 (60.24)	102 (45.33)	
BMI, kg/m^2^	21.5 (19.1, 24.2)	21.4 (19.0, 23.9)	22.0 (19.4, 24.6)	0.016
Hb, g/L	129 (116, 141)	130 (118, 142)	123 (109, 136)	< 0.001
RBC, × 10^9^/L	4.27 (3.85, 4.66)	4.30 (3.93, 4.68)	4.03 (3.64, 4.54)	< 0.001
WBC, × 10^9^/L	7.3 (5.8, 9.3)	7.3 (5.9, 9.3)	7.2 (5.4, 10.0)	0.751
PLT, × 10^9^/L	218 (167, 270)	225 (181, 278)	170 (132, 223)	< 0.001
N, × 10^9^/L	5.0 (3.7, 6.9)	4.9 (3.6, 6.8)	5.1 (3.7, 7.7)	0.330
L, × 10^9^/L	1.32 (0.90, 1.78)	1.38 (0.99, 1.81)	0.99 (0.69, 1.49)	< 0.001
M, × 10^9^/L	0.58 (0.42, 0.81)	0.58 (0.42, 0.81)	0.57 (0.40, 0.77)	0.492
ALT, U/L	16 (12, 25)	16 (12, 24)	17 (12, 27)	0.295
AST, U/L	23 (18, 30)	22 (18, 29)	24 (19, 34)	0.021
ALB, g/L	36.4 (32.7, 39.7)	37.0 (33.1, 40.3)	35.2 (31.9, 37.8)	< 0.001
BUN, mmol/L	6.1 (4.8, 7.9)	5.9 (4.7, 7.5)	7.1 (5.5, 9.5)	< 0.001
Cr, μmol/L	76 (64, 92)	75 (63, 89)	85 (67, 108)	< 0.001
UA, μmol/L	325 (256, 400)	317 (253, 386)	362 (267, 446)	< 0.001
TC, mmol/L	4.41 (3.64, 5.13)	4.55 (3.80, 5.22)	3.83 (3.14, 4.61)	< 0.001
LDL-C, mmol/L	2.46 (1.86, 3.04)	2.52 (1.95, 3.12)	2.11 (1.53, 2.83)	< 0.001
HDL-C, mmol/L	1.09 (0.90, 1.33)	1.10 (0.90, 1.34)	1.09 (0.92, 1.32)	0.677
Random glucose, mmol/L	6.30 (5.20, 7.90)	6.30 (5.10, 7.85)	6.40 (5.27, 8.40)	0.224
LVEF %	65 (60, 68)	65 (62, 68)	62 (56, 66)	< 0.001
SIRI	2.1 (1.2, 4.0)	2.0 (1.2, 3.7)	2.5 (1.4, 5.5)	< 0.001
Standardized SIRI	−0.31 (−0.47, 0.04)	−0.33 (−0.48, −0.02)	−0.24 (−0.43, 0.32)	< 0.001
**SIRI quartiles**				
Q1	282 (24.89)	239 (26.32)	43 (19.11)	< 0.001
Q2	284 (25.07)	236 (25.99)	48 (21.33)	
Q3	283 (24.98)	230 (25.33)	53 (23.56)	
Q4	284 (25.07)	203 (22.36)	81 (36.00)	

AF, atrial fibrillation; BMI, body mass index; WBC, white blood cell count; Hb, hemoglobin; RBC, red blood cell; WBC, white blood cell; PLT, platelet; N, neutrophil; L, lymphocyte; M, monocyte; ALT, alanine aminotransferase; AST, aspartate aminotransferase; ALB, albumin; BUN, blood urea nitrogen; Cr, creatinine; UA, uric acid; TC, total cholesterol; LDL-C, low-density lipoprotein cholesterol; HDL-C, high-density lipoprotein cholesterol; LVEF, left ventricular ejection fraction; SIRI, systemic inflammation response index.

### 3.2 Baseline characteristics of patients stratified by SIRI quartiles

Table 2 presents the baseline demographic and clinical characteristics of participants stratified by quartiles of SIRI. Compared with the Q1 group, patients in the Q4 group were older, had a higher proportion of males, and exhibited significantly elevated levels of WBC, PLT, N, M, AST, BUN, HDL-C, and random glucose (all *p* < 0.05). Additionally, the prevalence of heart failure, cerebrovascular disease, and pulmonary hypertension was higher, while BMI, Hb, RBC, L, ALB, UA, TC, and LDL-C levels, as well as the prevalence of malignancy, were lower in the Q4 group (all *p* < 0.05). Notably, the incidence of AF was significantly higher in the Q4 group (28.52 vs. 15.25%, *p* < 0.001).

**Table 2:** Baseline characteristics of patients stratified by SIRI quartiles.

**Variables**	**Total population**	**Quartiles of SIRI**				***p*-value**
	***N*** = **1,133**	**Q1**	**Q2**	**Q3**	**Q4**	
		***N*** = **282**	***N*** = **284**	***N*** = **283**	***N*** = **284**	
Age, years	73 (67, 81)	71 (64, 79)	74 (67, 80)	74 (67, 81)	76 (69, 82)	< 0.001
**Sex**, ***n*** **(%)**						
Male	973 (85.88)	225 (79.79)	245 (86.27)	250 (88.34)	253 (89.08)	0.006
Female	160 (14.12)	57 (20.21)	39 (13.73)	33 (11.66)	31 (10.92)	
**Smoking**, ***n*** **(%)**						
Yes	667 (58.87)	157 (55.67)	156 (54.93)	183 (64.66)	171 (60.21)	0.067
No	466 (41.13)	125 (44.33)	128 (45.07)	100 (35.34)	113 (39.79)	
**Drinking**, ***n*** **(%)**						
Yes	407 (35.92)	87 (30.85)	106 (37.32)	105 (37.10)	109 (38.38)	0.230
No	726 (64.08)	195 (69.15)	178 (62.68)	178 (62.90)	175 (61.62)	
**Asthma**, ***n*** **(%)**						
Yes	31 (2.74)	11 (3.90)	10 (3.52)	4 (1.41)	6 (2.11)	0.224
No	1,102 (97.26)	271 (96.10)	274 (96.48)	279 (98.59)	278 (97.89)	
**Heart failure**, ***n*** **(%)**						
Yes	144 (12.71)	26 (9.22)	34 (11.97)	33 (11.66)	51 (17.96)	0.014
No	989 (87.29)	256 (90.78)	250 (88.03)	250 (88.34)	233 (82.04)	
**Hypertension**, ***n*** **(%)**						
Yes	453 (39.98)	109 (38.65)	132 (46.48)	106 (37.46)	106 (37.32)	0.079
No	680 (60.02)	173 (61.35)	152 (53.52)	177 (62.54)	178 (62.68)	
**Diabetes**, ***n*** **(%)**						
Yes	165 (14.56)	40 (14.18)	37 (13.03)	42 (14.84)	46 (16.20)	0.754
No	968 (85.44)	242 (85.82)	247 (86.97)	241 (85.16)	238 (83.80)	
**Cerebrovascular disease**, ***n*** **(%)**						
Yes	124 (10.94)	19 (6.74)	31 (10.92)	35 (12.37)	39 (13.73)	0.047
No	1,009 (89.06)	263 (93.26)	253 (89.08)	248 (87.63)	245 (86.27)	
**Coronary heart disease**, ***n*** **(%)**						
Yes	113 (9.97)	21 (7.45)	29 (10.21)	36 (12.72)	27 (9.51)	0.215
No	1,020 (90.03)	261 (92.55)	255 (89.79)	247 (87.28)	257 (90.49)	
**Malignant tumors**, ***n*** **(%)**						
Yes	258 (22.77)	76 (26.95)	79 (27.82)	63 (22.26)	40 (14.08)	< 0.001
No	875 (77.23)	206 (73.05)	205 (72.18)	220 (77.74)	244 (85.92)	
**Pulmonary hypertension**, ***n*** **(%)**						
Yes	484 (42.72)	98 (34.75)	114 (40.14)	119 (42.05)	153 (53.87)	< 0.001
No	649 (57.28)	184 (65.25)	170 (59.86)	164 (57.95)	131 (46.13)	
BMI, kg/m^2^	21.5 (19.1, 24.2)	21.9 (19.5, 24.3)	21.7 (19.5, 24.4)	21.5 (19.1, 24.0)	20.4 (18.0, 23.5)	< 0.001
Hb, g/L	129 (116, 141)	133 (120, 144)	130 (118, 142)	127 (111, 141)	126 (112, 137)	< 0.001
RBC, × 10^9^/L	4.27 (3.85, 4.66)	4.32 (3.95, 4.69)	4.37 (3.89, 4.71)	4.21 (3.78, 4.63)	4.16 (3.78, 4.59)	0.006
WBC, × 10^9^/L	7.3 (5.8, 9.3)	5.5 (4.5, 6.5)	6.8 (5.7, 8.1)	7.9 (6.7, 9.2)	10.6 (8.7, 13.6)	< 0.001
PLT, × 10^9^/L	218 (167, 270)	209 (163, 251)	222 (170, 269)	225 (177, 277)	215 (166, 276)	0.008
N, × 10^9^/L	5.0 (3.7, 6.9)	3.2 (2.6, 3.9)	4.4 (3.7, 5.3)	5.7 (4.7, 6.8)	8.4 (6.7, 11.4)	< 0.001
L, × 10^9^/L	1.32 (0.90, 1.78)	1.61 (1.22, 2.04)	1.48 (1.08, 1.92)	1.29 (0.90, 1.69)	0.95 (0.64, 1.35)	< 0.001
M, × 10^9^/L	0.58 (0.42, 0.81)	0.40 (0.31, 0.50)	0.53 (0.43, 0.67)	0.64 (0.50, 0.81)	0.87 (0.64, 1.09)	< 0.001
ALT, U/L	16 (12, 25)	18 (12, 25)	15 (11, 23)	15 (11, 24)	18 (12, 28)	0.003
AST, U/L	23 (18, 30)	23 (19, 30)	22 (18, 30)	21 (17, 28)	24 (18, 32)	0.006
ALB, g/L	36.4 (32.7, 39.7)	38.3 (35.1, 41.1)	37.9 (35.0, 40.5)	35.7 (32.5, 38.7)	33.5 (30.3, 37.4)	< 0.001
BUN, mmol/L	6.1 (4.8, 7.9)	5.9 (4.7, 7.2)	6.2 (4.8, 7.8)	5.9 (4.6, 7.7)	6.8 (5.2, 9.3)	< 0.001
Cr, μmol/L	76 (64, 92)	74 (63, 87)	77 (66, 93)	77 (64, 93)	76 (62, 97)	0.150
UA, μmol/L	325 (256, 400)	332 (272, 406)	344 (277, 412)	323 (255, 403)	292 (214, 378)	< 0.001
TC, mmol/L	4.41 (3.64, 5.13)	4.59 (3.86, 5.26)	4.53 (3.89, 5.19)	4.35 (3.53, 5.13)	4.21 (3.43, 4.96)	< 0.001
LDL-C, mmol/L	2.46 (1.86, 3.04)	2.61 (1.98, 3.25)	2.56 (2.06, 3.09)	2.39 (1.83, 2.99)	2.23 (1.77, 2.88)	< 0.001
HDL-C, mmol/L	1.09 (0.90, 1.33)	1.11 (0.92, 1.31)	1.11 (0.94, 1.34)	1.05 (0.86, 1.27)	1.13 (0.89, 1.39)	0.040
Random glucose, mmol/L	6.30 (5.20, 7.90)	6.00 (4.90, 7.50)	6.20 (5.20, 7.55)	6.30 (5.20, 8.00)	6.70 (5.40, 8.56)	< 0.001
LVEF %	65 (60, 68)	65 (61, 68)	65 (61, 68)	65 (60, 67)	65 (60, 67)	0.139
SIRI	2.1 (1.2, 4.0)	0.8 (0.7, 1.0)	1.6 (1.4, 1.8)	2.8 (2.4, 3.3)	6.8 (5.2, 10.7)	< 0.001
Standardized SIRI	−0.31 (−0.47, 0.04)	−0.54 (−0.58, −0.50)	−0.40 (−0.44, −0.36)	−0.19 (−0.25, −0.09)	0.58 (0.26, 1.29)	< 0.001
**Atrial fibrillation**, ***n*** **(%)**						
Yes	225 (19.86)	43 (15.25)	48 (16.90)	53 (18.73)	81 (28.52)	< 0.001
No	908 (80.14)	239 (84.75)	236 (83.10)	230 (81.27)	203 (71.48)	

SIRI, systemic inflammation response index; BMI, body mass index; WBC, white blood cell count; Hb, hemoglobin; RBC, red blood cell; WBC, white blood cell; PLT, platelet; N, neutrophil; L, lymphocyte; M, monocyte; ALT, alanine aminotransferase; AST, aspartate aminotransferase; ALB, albumin; BUN, blood urea nitrogen; Cr, creatinine; UA, uric acid; TC, total cholesterol; LDL-C, low-density lipoprotein cholesterol; HDL-C, high-density lipoprotein cholesterol; LVEF, left ventricular ejection fraction.

### 3.3 Association between SIRI and atrial fibrillation

As shown in Table 3, univariate logistic regression analysis indicated that age, smoking, asthma, heart failure, hypertension, cerebrovascular disease, coronary artery disease, malignancy, pulmonary hypertension, BMI, Hb, RBC, PLT, ALB, BUN, Cr, UA, TC, LDL-C, LVEF, SIRI were all significantly associated with AF (*p* < 0.05). As shown in Table 4, multivariate logistic regression analysis revealed that an increase in SIRI levels was significantly associated with a higher risk of AF in both Model 1 and Model 2, regardless of whether SIRI was treated as a continuous or categorical variable (*p* < 0.05). In Model 3, which adjusted for all confounding factors, patients in the Q4 group exhibited a significant 116.2% increase in the risk of atrial fibrillation (AF) compared to the Q1 group (OR = 2.162, 95% CI: 1.325–3.527, *p* = 0.002).

**Table 3:** Univariate logistic regression analysis for atrial fibrillation.

**Variables**	**OR**	**95% CI**	***p*-value**
Age, years	1.089	1.069, 1.110	< 0.001
**Sex**			
Female	Ref		
Male	0.764	0.513, 1.137	0.184
Smoking	0.738	0.550, 0.989	0.042
Drinking	0.957	0.705, 1.299	0.777
Asthma	0.131	0.018, 0.961	0.046
Heart failure	4.417	3.054, 6.389	< 0.001
Hypertension	1.541	1.148, 2.067	0.004
Diabetes	1.010	0.669, 1.527	0.961
Cerebrovascular disease	3.780	2.556, 5.591	< 0.001
Coronary heart disease	2.056	1.342, 3.148	< 0.001
Malignant tumors	0.660	0.452, 0.962	0.031
Pulmonary hypertension	1.827	1.361, 2.452	< 0.001
BMI	1.044	1.006, 1.083	0.022
Hb	0.984	0.977, 0.990	< 0.001
RBC	0.619	0.497, 0.772	< 0.001
WBC	1.026	0.997, 1.056	0.079
PLT	0.992	0.989, 0.994	< 0.001
ALT	1.002	1.000, 1.005	0.101
AST	1.002	0.999, 1.004	0.268
ALB	0.936	0.910, 0.963	< 0.001
BUN	1.087	1.052, 1.123	< 0.001
Cr	1.002	1.000, 1.004	0.012
UA	1.003	1.002, 1.004	< 0.001
TC	0.623	0.540, 0.717	< 0.001
LDL-C	0.636	0.530, 0.763	< 0.001
HDL-C	0.837	0.542, 1.291	0.420
Random glucose	1.031	0.983, 1.083	0.210
LVEF	0.945	0.929, 0.962	< 0.001
SIRI	1.050	1.025, 1.075	< 0.001
Standardized SIRI	1.031	0.983, 1.083	< 0.001
**SIRI quartiles**			
Q1	Ref		
Q2	1.130	0.721, 1.771	0.593
Q3	1.281	0.824, 1.991	0.272
Q4	2.218	1.465, 3.357	< 0.001

BMI, body mass index; WBC, white blood cell count; Hb, hemoglobin; RBC, red blood cell; WBC, white blood cell; PLT, platelet; ALT, alanine aminotransferase; AST, aspartate aminotransferase; ALB, albumin; BUN, blood urea nitrogen; Cr, creatinine; UA, uric acid; TC, total cholesterol; LDL-C, low-density lipoprotein cholesterol; HDL-C, high-density lipoprotein cholesterol; LVEF, left ventricular ejection fraction.

**Table 4:** Multivariate logistic regression analysis of SIRI and atrial fibrillation.

**Variables**	**Model 1**		**Model 2**		**Model 3**	
	**OR (95% CI)**	***p*** **-value**	**OR (95% CI)**	***p*** **-value**	**OR (95% CI)**	***p*** **-value**
SIRI	1.045 (1.019, 1.071)	0.001	1.041 (1.014, 1.069)	0.003	1.046 (1.018, 1.075)	0.001
Standardized SIRI	1.265 (1.108, 1.443)	0.001	1.242 (1.077, 1.433)	0.003	1.274 (1.100, 1.474)	0.001
**SIRI quartiles**						
Q1	Ref		Ref		Ref	
Q2	0.973 (0.610, 1.553)	0.910	0.908 (0.558, 1.477)	0.697	1.072 (0.644, 1.785)	0.788
Q3	1.161 (0.734, 1.838)	0.523	1.102 (0.682, 1.781)	0.691	1.297 (0.782, 2.150)	0.314
Q4	1.818 (1.179, 2.803)	0.007	1.696 (1.073, 2.682)	0.024	2.162 (1.325, 3.527)	0.002
*p* for trend		0.001		0.003		< 0.001

Model 1: adjusted for age.

Model 2: adjusted for age, smoking, asthma, heart failure, hypertension, cerebrovascular disease, coronary heart disease, malignant tumor, pulmonary hypertension, BMI; Model 3: Model2 plus further adjusted for Hb, RBC, PLT, ALB, BUN, Cr, UA, TC, LDL-C, LVEF.

OR, odds ratio; CI, confidence interval; SIRI, systemic inflammation response index; BMI, body mass index; Hb, hemoglobin; RBC, red blood cell; PLT, platelet count; ALB, albumin; BUN, blood urea nitrogen; Cr, creatinine; UA, uric acid; TC, total cholesterol; LDL-C, low-density lipoprotein cholesterol; LVEF, left ventricular ejection fraction.

**Figure 2:**
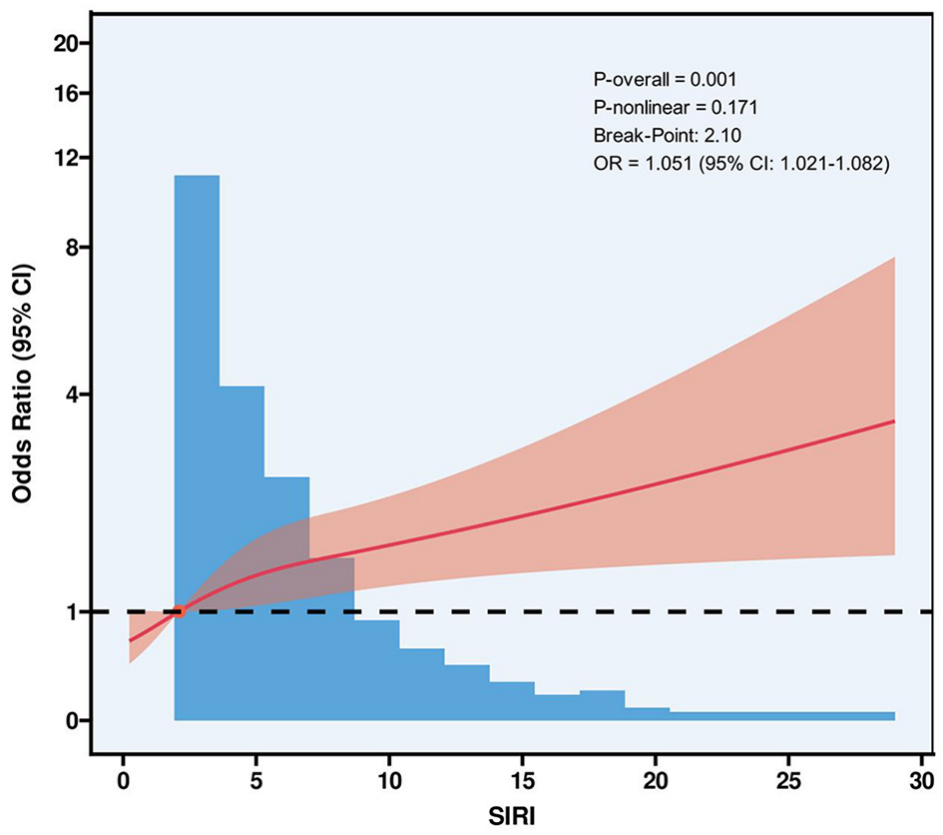
The RCS plot describes the association between SIRI and the risk of AF. The model was conducted with four knots at the 5th, 35th, 65th, and 95th percentiles of SIRI (reference is the median). It was adjusted for all covariates in Table 4. SIRI, systemic inflammation response index; AF, atrial fibrillation; OR, odds ratio.

In Model 3, after full adjustment for potential confounders, higher SIRI levels remained significantly associated with an increased risk of AF. Specifically, individuals in the Q4 group had a 116.2% higher risk of AF compared to the Q1 group (OR = 2.162, 95% CI: 1.325–3.527, *p* = 0.002). Trend analysis further revealed a significant dose–dependent increase in AF risk across ascending SIRI quartiles (*p* for trend < 0.05). When SIRI was analyzed as a continuous variable, each one-unit and one-standard deviation increase in SIRI was associated with a 4.6 and 27.4% increased risk of AF, respectively (SIRI: OR = 1.046, 95% CI: 1.018–1.075, *p* = 0.002; standardized SIRI: OR = 1.274, 95% CI: 1.100–1.474, *p* = 0.002). Furthermore, RCS regression analysis demonstrated a significant linear dose-response relationship between SIRI and the risk of AF (*p* for non-linearity > 0.05), with the risk of AF rising continuously as SIRI levels increased (*p* for overall trend < 0.05; Figure 2).

### 3.4 Predictive value of SIRI for atrial fibrillation

**Figure 3:**
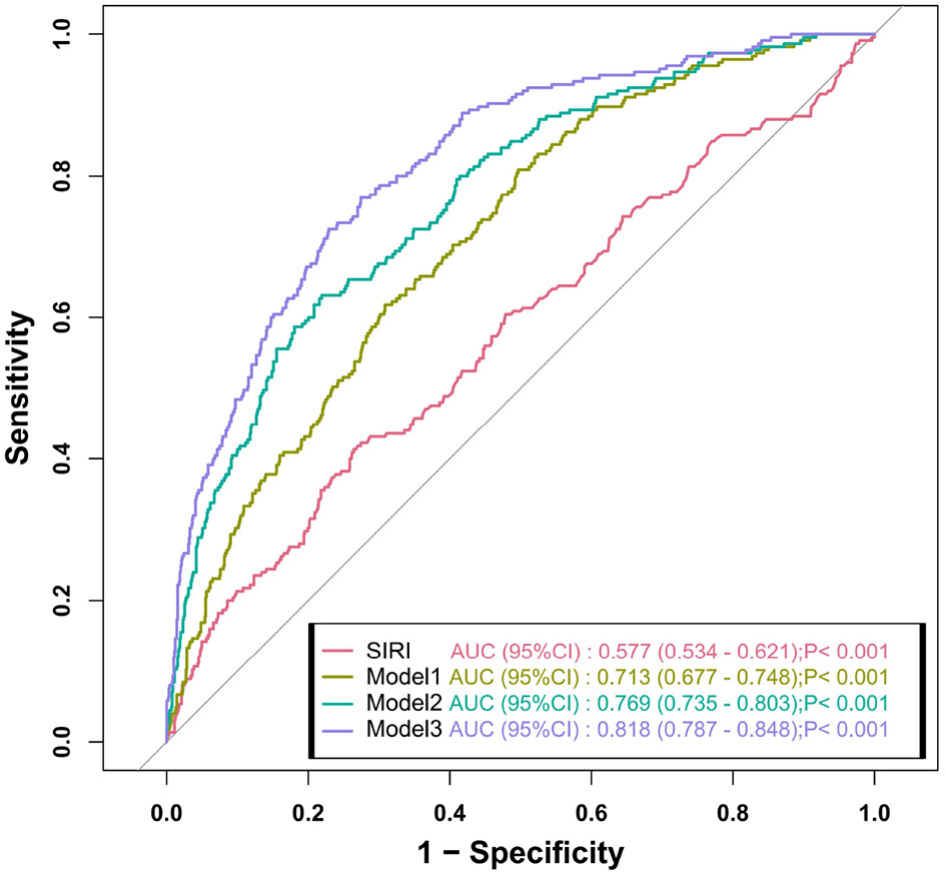
Receiver operating characteristic analysis comparing the predictive ability of three logistic regression models. Adjusted covariates: Model 1: age; Model 2: age, smoking, asthma, heart failure, hypertension, cerebrovascular disease, coronary heart disease, malignant tumor, pulmonary hypertension, BMI; Model 3: Model 2 + Hb, RBC, PLT, ALB, BUN, Cr, UA, TC, LDL-C, LVEF. BMI, body mass index; Hb, hemoglobin; RBC, red blood cell; PLT, platelet count; ALB, albumin; BUN, blood urea nitrogen; Cr, creatinine; UA, uric acid; TC, total cholesterol; LDL-C, low-density lipoprotein cholesterol; LVEF, left ventricular ejection fraction.

To evaluate the predictive value of SIRI for the risk of AF in patients with COPD, and to compare its performance against different logistic regression models, we performed ROC analysis. As shown in Figure 3, the AUC for the SIRI was 0.577 (95% CI: 0.534–0.621, *p* < 0.001), indicating a certain discriminatory ability in predicting the risk of AF. In comparison, Model 1 yielded an AUC of 0.713 (95% CI: 0.677–0.748, *p* < 0.001); Model 2 achieved an AUC of 0.769 (95% CI: 0.735–0.803, *p* < 0.001); and Model 3 demonstrated the best performance, with an AUC of 0.818 (95% CI: 0.787–0.848, *p* < 0.001). The results indicate that although SIRI alone may serve as an independent predictor of AF, the predictive performance was significantly improved when additional clinical risk factors were considered.

### 3.5 Subgroup and interaction analyses

**Figure 4:**
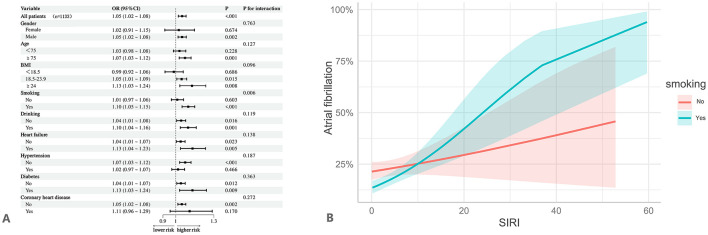
Subgroup and interaction analyses. It was adjusted for all covariates in Table 4. **(A)** Subgroup and interaction analyses for the association between SIRI and atrial fibrillation. **(B)** Interaction between SIRI and smoking on atrial fibrillation.

As shown in Table 5, individuals in the Q4 group had a significantly increased risk of AF compared to those in the Q1 group, particularly in the following subgroups: age ≥ 75 years, male, BMI ≥ 24, smoking, non-drinking, heart failure, hypertension, diabetes, without coronary heart disease (*p* < 0.05). Notably, both elevated SIRI and standardized SIRI levels were significantly associated with a higher risk of AF in the following subgroups: age ≥75 years, male, drinking, BMI ≥ 18.5, smoking, non-drinking, individuals with or without heart failure, with or without diabetes, without hypertension, and without coronary heart disease (*p* < 0.05). Furthermore, interaction analyses demonstrated that the positive association between SIRI and AF remained consistent across most subgroups stratified by age, sex, BMI, drinking, heart failure, hypertension, diabetes, and coronary heart disease (all interaction *p*-values > 0.05), indicating that the association is robust overall (Figure 4A). However, a significant interaction was observed in the smoking subgroup. With increasing SIRI levels, the risk of AF rose sharply among smokers, suggesting that smoking may amplify the association between SIRI and AF (OR = 1.10, 95% CI: 1.05–1.15, *p* < 0.001; interaction *p* = 0.006; Figure 4B).

**Table 5:** Subgroup analysis for the association between SIRI and atrial fibrillation.

**Subgroups**	**Q2 vs. Q1**		**Q3 vs. Q1**		**Q4 vs. Q1**		**SIRI**		**Standardized SIRI**	
	**OR (95% CI)**	***p*** **-value**	**OR (95% CI)**	***p*** **-value**	**OR (95% CI)**	***p*** **-value**	**OR (95% CI)**	***p*** **-value**	**OR (95% CI)**	***p*** **-value**
**Age**								
< 75 years	0.530 (0.222, 1.266)	0.153	0.993 (0.429, 2.296)	0.986	1.750 (0.784, 3.908)	0.172	1.029 (0.980, 1.080)	0.246	1.165 (0.900, 1.510)	0.246
≥75 years	1.344 (0.674, 2.681)	0.401	1.450 (0.740, 2.841)	0.279	2.188 (1.144, 4.183)	0.018	1.071 (1.028, 1.115)	0.001	1.442 (1.158, 1.795)	0.001
**Sex**								
Male	1.042 (0.595, 1.825)	0.885	1.148 (0.656, 2.011)	0.629	1.978 (1.160, 3.373)	0.012	1.046 (1.016, 1.077)	0.002	1.274 (1.091, 1.488)	0.002
Female	0.567 (0.090, 3.548)	0.544	0.952 (0.157, 5.788)	0.957	3.391 (0.491, 23.422)	0.216	1.044 (0.929, 1.172)	0.470	1.258 (0.675, 2.345)	0.470
**BMI**								
< 18.5	0.597 (0.100, 3.569)	0.572	1.160 (0.246, 5.477)	0.851	1.114 (0.250, 4.960)	0.888	0.985 (0.913, 1.063)	0.698	0.922 (1.613, 1.388)	0.698
18.5–23.9	0.993 (0.495, 1.991)	0.983	1.098 (0.543, 2.221)	0.796	1.989 (0.977, 4.049)	0.058	1.048 (1.013, 1.084)	0.007	1.287 (1.073, 1.544)	0.007
≥24	1.635 (0.613, 4.359)	0.326	1.468 (0.535, 4.027)	0.456	4.453 (1.685, 11.772)	0.003	1.125 (1.034, 1.224)	0.006	1.878 (1.197, 2.947)	0.006
**Smoking**								
Yes	0.768 (0.374, 1.576)	0.471	1.022 (0.521, 2.006)	0.949	2.234 (1.169, 4.269)	0.015	1.088 (1.044, 1.134)	< 0.001	1.571 (1.256, 1.964)	< 0.001
No	1.374 (0.605, 3.119)	0.448	1.594 (0.672, 3.779)	0.290	2.159 (0.920, 5.063)	0.077	1.013 (0.966, 1.063)	0.596	1.072 (0.830, 1.384)	0.596
**Drinking**								
Yes	0.736 (0.297, 1.824)	0.508	1.152 (0.469, 2.830)	0.758	2.417 (0.957, 6.102)	0.062	1.083 (1.027, 1.141)	0.003	1.531 (1.155, 2.029)	0.003
No	1.349 (0.691, 2.631)	0.380	1.297 (0.674, 2.497)	0.436	2.555 (1.323, 4.933)	0.005	1.035 (1.002, 1.069)	0.039	1.200 (1.009, 1.427)	0.039
**Heart failure**								
Yes	2.505 (0.659, 9.513)	0.178	2.309 (0.589, 9.058)	0.230	6.979 (2.008, 24.259)	0.002	1.136 (1.040, 1.241)	0.005	1.981 (1.234, 3.178)	0.005
No	0.845 (0.466, 1.533)	0.580	1.140 (0.635, 2.046)	0.660	1.602 (0.872, 2.946)	0.129	1.042 (1.009, 1.077)	0.012	1.249 (1.051, 1.485)	0.012
**Hypertension**								
Yes	1.747 (0.802, 3.807)	0.160	1.757 (0.788, 3.919)	0.169	2.559 (1.139, 5.749)	0.023	1.018 (0.968, 1.071)	0.482	1.102 (0.840, 1.447)	0.482
No	0.729 (0.331, 1.605)	0.432	1.053 (0.508, 2.182)	0.889	2.545 (1.218, 5.317)	0.013	1.074 (1.032, 1.117)	< 0.001	1.464 (1.181, 1.813)	< 0.001
**Diabetes**								
Yes	2.535 (0.552, 11.636)	0.232	1.377 (0.280, 6.760)	0.694	6.962 (1.767, 27.435)	0.006	1.108 (1.034, 1.187)	0.004	1.732 (1.195, 2.510)	0.004
No	0.827 (0.466, 1.465)	0.515	1.175 (0.668, 2.067)	0.575	1.772 (0.995, 3.156)	0.052	1.039 (1.008, 1.070)	0.012	1.227 (1.046, 1.440)	0.012
**Coronary heart disease**								
Yes	2.612 (0.464, 14.710)	0.276	1.623 (0.282, 9.328)	0.587	3.320 (0.477, 23.103)	0.225	1.119 (0.959, 1.305)	0.152	1.825 (0.801, 4.158)	0.152
No	0.955 (0.546, 1.673)	0.873	1.387 (0.796, 2.414)	0.248	1.878 (1.101, 3.204)	0.021	1.043 (1.013, 1.073)	0.004	1.251 (1.074, 1.457)	0.004

SIRI, systemic inflammation response index; OR, odds ratio; CI, confidence interval; BMI, body mass index.

### 3.6 Sensitivity and robustness analyses

Sensitivity analyses confirmed the robustness of the association between the SIRI and AF across different stratification methods, including tertiles, median, and mean values. In the fully adjusted Model 3, individuals in the highest SIRI tertile (T3) had a significantly increased risk of AF (OR = 1.904, 95% CI: 1.239–2.923, *p* = 0.003). Similarly, elevated risks were observed in the high SIRI group when stratified by median (OR = 1.569, 95% CI: 1.110–2.219, *p* = 0.011) and mean values (OR = 1.886, 95% CI: 1.305–2.727, *p* = 0.001). All trend tests were statistically significant (*p* for trend < 0.05), supporting a positive dose-response relationship between increasing SIRI levels and AF risk. Notably, this association remained significant even after excluding patients with asthma (continuous SIRI: OR = 1.045, 95% CI: 1.017–1.074, *p* = 0.002; standardized SIRI: OR = 1.266, 95% CI: 1.094–1.464, *p* = 0.002; Q4 vs. Q1: OR = 2.065, 95% CI: 1.260–3.384, *p* = 0.004). Similar associations were observed after excluding patients with cerebrovascular disease, malignancies, or pulmonary hypertension, with all models consistently demonstrating statistically significant trends (*p* for trend < 0.05 across all exclusion analyses; detailed results are presented in Table 6).

**Table 6:** Multivariate logistic regression of systemic inflammation response index and atrial fibrillation: sensitivity analyses.

**Variables**	**Model 1**		**Model 2**		**Model 3**	
	**OR (95% CI)**	***p*** **-value**	**OR (95% CI)**	***p*** **-value**	**OR (95% CI)**	***p*** **-value**
**By tertiles**						
T1	Ref		Ref		Ref	
T2	1.121 (0.753, 1.667)	0.574	1.077 (0.712, 1.629)	0.726	1.224 (0.791, 1.894)	0.365
T3	1.662 (1.138, 2.427)	0.009	1.623 (1.087, 2.422)	0.018	1.904 (1.239, 2.923)	0.003
*p* for trend		0.004		0.009		0.002
**By median split**						
Low SIRI	Ref		Ref		Ref	
High SIRI	1.503 (1.104, 2.048)	0.010	1.460 (1.054, 2.023)	0.023	1.569 (1.110, 2.219)	0.011
**By mean split**						
Low SIRI	Ref		Ref		Ref	
High SIRI	1.704 (1.233, 2.356)	0.001	1.682 (1.192, 2.374)	0.003	1.886 (1.305, 2.727)	0.001
**Exclusion of people with asthma**						
SIRI	1.044 (1.018, 1.070)	0.001	1.041 (1.013, 1.069)	0.003	1.045 (1.017, 1.074)	0.002
Standardized SIRI	1.258 (1.102, 1.436)	0.001	1.238 (1.073, 1.428)	0.003	1.266 (1.094, 1.464)	0.002
**SIRI quartiles**						
Q1	Ref		Ref		Ref	
Q2	0.985 (0.616, 1.575)	0.950	0.922 (0.566, 1.502)	0.745	1.095 (0.655, 1.830)	0.730
Q3	1.147 (0.724, 1.816)	0.559	1.094 (0.677, 1.769)	0.713	1.239 (0.743, 2.063)	0.411
Q4	1.776 (1.150, 2.744)	0.010	1.674 (1.058, 2.650)	0.028	2.065 (1.260, 3.384)	0.004
*p* for trend		0.001		0.004		0.001
**Exclusion of people with cerebrovascular disease**						
SIRI	1.049 (1.019, 1.080)	0.001	1.044 (1.014, 1.075)	0.004	1.043 (1.012, 1.075)	0.007
Standardized SIRI	1.291 (1.107, 1.507)	0.001	1.258 (1.076, 1.470)	0.003	1.254 (1.065, 1.476)	0.007
**SIRI quartiles**						
Q1	Ref		Ref		Ref	
Q2	0.857 (0.504, 1.458)	0.568	0.834 (0.481, 1.443)	0.516	0.922 (0.522, 1.631)	0.781
Q3	1.157 (0.693, 1.931)	0.577	1.214 (0.716, 2.058)	0.472	1.243 (0.715, 2.163)	0.441
Q4	2.005 (1.243, 3.233)	0.004	1.962 (1.189, 3.238)	0.008	2.203 (1.304, 3.721)	0.003
*p* for trend		< 0.001		< 0.001		< 0.001
**Exclusion of people with malignant tumors**						
SIRI	1.046 (1.018, 1.074)	0.001	1.046 (1.016, 1.076)	0.002	1.053 (1.022, 1.085)	0.001
Standardized SIRI	1.270 (1.101, 1.464)	0.001	1.271 (1.090, 1.482)	0.002	1.320 (1.126, 1.547)	0.001
**SIRI quartiles**						
Q1	Ref		Ref		Ref	
Q2	1.005 (0.590, 1.714)	0.984	0.914 (0.523, 1.598)	0.753	1.115 (0.610, 2.038)	0.723
Q3	1.072 (0.636, 1.806)	0.794	1.022 (0.592, 1.766)	0.936	1.197 (0.662, 2.163)	0.552
Q4	1.736 (1.071, 2.812)	0.025	1.720 (1.031, 2.869)	0.038	2.281 (1.300, 4.004)	0.004
*p* for trend		0.005		0.005		0.001
SIRI	1.099 (1.052, 1.148)	< 0.001	1.086 (1.041, 1.133)	< 0.001	1.073 (1.023, 1.126)	0.004
Standardized SIRI	1.657 (1.310, 2.095)	< 0.001	1.555 (1.241, 1.949)	< 0.001	1.462 (1.131, 1.889)	0.004
**SIRI quartiles**						
Q1	Ref		Ref		Ref	
Q2	0.920 (0.459, 1.847)	0.815	0.853 (0.417, 1.743)	0.662	1.065 (0.493, 2.304)	0.872
Q3	1.357 (0.702, 2.622)	0.364	1.171 (0.590, 2.324)	0.652	1.576 (0.738, 3.365)	0.240
Q4	3.339 (1.795, 6.213)	< 0.001	2.616 (1.361, 5.028)	0.004	3.904 (1.872, 8.141)	< 0.001
*p* for trend		< 0.001		< 0.001		< 0.001

OR, odds ratio; CI, confidence interval; SIRI, systemic inflammation response index.

## 4 Discussion

This study is the first to systematically evaluate the association between the SIRI and the risk of AF in patients with COPD. Our findings demonstrate that elevated SIRI levels are significantly associated with an increased risk of AF. This association remained robust even after stepwise adjustment for all potential confounders. Trend analysis and RCS modeling further revealed a significant linear dose–response relationship between SIRI and AF risk. Moreover, ROC curve analysis demonstrated that SIRI exhibits a moderate predictive ability for AF, and that a prediction model incorporating SIRI alongside other risk factors significantly improves the overall predictive performance. Subgroup and interaction analyses further confirmed a consistent positive association between SIRI and the risk of AF across different subgroups. Multiple sensitivity analyses—such as applying alternative stratification criteria and excluding key comorbidities—further confirmed the robustness and reliability of our findings. Taken together, these results suggest that SIRI, as a simple, stable, and integrative marker of systemic inflammation, may serve as a valuable tool for early identification and risk stratification of AF in patients with COPD. These findings may provide important insights for the development of individualized prevention and intervention strategies in this high-risk population.

Based on its simplicity and stability, SIRI holds significant promise for clinical translation in COPD patients. Its ease of calculation during standard blood work positions it as a low-cost, readily available screening tool; elevated levels could flag high-risk individuals, prompting intensified cardiovascular monitoring (e.g., more frequent ECGs or Holter monitoring) to facilitate earlier AF detection and intervention. Beyond screening, SIRI could refine risk stratification by complementing established clinical factors (e.g., age, COPD severity/GOLD stage, comorbidities like heart failure, hypertension, diabetes) within future COPD-specific AF risk models. This integration aims to enhance predictive accuracy, identify patients most likely to benefit from aggressive risk factor modification (e.g., blood pressure/glycemic control, smoking cessation), and potentially guide personalized management decisions. While SIRI directly reflects a key inflammatory pathway linking COPD to AF, prospective validation is needed to determine optimal thresholds and its incremental value over existing models.

In recent years, an increasing body of evidence has suggested that the SIRI plays a significant role in the pathogenesis of AF and in predicting its risk. For instance, Lin et al. (15) reported in a retrospective study involving patients with ischemic stroke that elevated SIRI levels were significantly associated with the presence of AF, and this association remained stable even after adjusting for multiple confounding factors, indicating the potential predictive value of SIRI in stroke populations. Similarly, Wang et al. (28) found that higher SIRI levels markedly increased the risk of new-onset AF in patients with ST-segment elevation myocardial infarction (STEMI), suggesting that SIRI may serve as an effective tool for risk stratification among patients with acute coronary syndromes. Moreover, Chi et al. (29) further revealed a close association between SIRI levels and left ventricular remodeling as well as systolic dysfunction in patients with AF, implying that systemic inflammation might not only contribute to the development of AF but also mediate structural and functional cardiac alterations. Meanwhile, Zhao et al. (30) focused on inflammatory profiles in patients with paroxysmal AF and demonstrated that these patients exhibited significantly elevated SIRI levels, supporting its potential utility as an auxiliary screening marker. In a prospective cohort study including AF patients with type 2 diabetes mellitus, Chen et al. (31) showed that elevated SIRI levels significantly increased the risk of long-term adverse cardiovascular events, highlighting its clinical significance in populations with multiple comorbidities. Furthermore, Luo et al. (32) systematically analyzed the relationships between blood inflammatory markers and AF, heart failure, and cardiovascular mortality in a large prospective cohort study, revealing that elevated SIRI levels significantly increased the risk of these cardiovascular adverse outcomes. Overall, current evidence consistently indicates a significant and stable association between SIRI and AF across diverse populations. However, to our knowledge, no studies have yet explored whether this association holds true in patients with COPD. Unlike previous studies, the present study is the first to investigate the relationship between SIRI and the risk of AF specifically in patients with COPD. Through subgroup analyses and multiple sensitivity analyses, we further confirmed the robustness and independence of this association. These findings provide novel insights and potential inflammatory biomarkers to support the early identification, and clinical management of AF risk in the COPD population.

Notably, our study identified a significant interaction between smoking and the SIRI. Smoking may amplify the association between the SIRI and AF, suggesting that exogenous inflammatory stimuli could synergize with the patient's intrinsic inflammatory status, thereby further increasing the risk of AF. This finding may be explained by the following mechanism: First, smoking, as a persistent source of exogenous inflammation, can trigger widespread immune activation and a state of chronic low-grade inflammation. Cigarette smoke contains numerous harmful components, such as reactive oxygen species, carbon monoxide, and nicotine, which directly irritate the respiratory mucosa and stimulate systemic immune responses. These stimuli promote the recruitment and activation of neutrophils and monocytes, leading to the release of large quantities of pro-inflammatory cytokines, including interleukin-6 (IL-6), tumor necrosis factor-alpha (TNF-α), and CRP (33, 34). Persistent release of these inflammatory mediators not only maintains a high level of systemic inflammation but may also accelerate structural and electrophysiological remodeling of the atria, creating a vulnerable substrate for AF development (35, 36). Second, smoking can suppress both the number and function of lymphocytes—particularly T cell subsets such as CD4+ T cells—resulting in decreased proliferative capacity and increased rates of apoptosis, which may further elevate SIRI levels (37). This dual state of inflammatory activation and immune suppression could be a key pathway underlying elevated SIRI in smokers. Furthermore, studies have demonstrated that smoking can influence epigenetic regulation and bone marrow hematopoiesis, promoting the differentiation of myeloid cells—including neutrophils and monocytes—toward pro-inflammatory phenotypes, while simultaneously inhibiting the development of lymphoid cell lineages (38). This leads to an immune profile characterized by heightened innate immunity and relatively suppressed adaptive immunity. Such immune dysregulation may exert sustained effects on the atrial microenvironment, contributing to inflammation-mediated atrial remodeling. Importantly, the systemic inflammatory state reflected by SIRI in smokers may already be in a pre-activated condition, potentially exerting more pronounced disturbances on cardiac electrophysiological homeostasis, even at preclinical stages (39). This low-threshold, high-responsiveness inflammatory background may enhance the predictive value of SIRI for atrial fibrillation (AF) in smokers, further supporting a synergistic pathogenic role between smoking and elevated SIRI levels (40, 41). In summary, smoking may significantly amplify the systemic inflammatory burden reflected by SIRI through mechanisms involving enhanced neutrophil and monocyte-driven inflammation, suppression of lymphocyte function, and remodeling of the immune microenvironment, thereby strengthening the association between SIRI and AF.

Although the findings of this study are of significant clinical interest, the underlying biological mechanisms remain to be fully elucidated. Elevated SIRI reflects a synergistic imbalance between heightened pro-inflammatory immunity, primarily driven by neutrophils and monocytes, and suppressed immune regulation mediated by lymphocytes. A review of relevant literature suggests that this immunological imbalance may play a critical role in the pathogenesis of AF. First, the two key pro-inflammatory cell types integrated within SIRI—neutrophils and monocytes—have both been implicated as central players in the pathophysiological mechanisms of AF. Neutrophils can release reactive oxygen species, myeloperoxidase, and form neutrophil extracellular traps, all of which contribute to localized oxidative stress and tissue injury within the atria. These processes promote both electrical and structural remodeling of the atrial substrate (41–43). Meanwhile, monocytes, upon migrating into atrial tissue, can differentiate into pro-inflammatory macrophages that secrete substantial amounts of cytokines such as interleukin-6 (IL-6) and tumor necrosis factor-alpha (TNF-α), as well as matrix metalloproteinases (MMPs). These mediators activate fibroblasts and drive atrial interstitial fibrosis, forming a pathological substrate conducive to AF development (44–46). Second, the reduction in lymphocyte count—reflected as the denominator in the SIRI calculation—has also been recognized as an indicator of increased susceptibility to AF. Lymphopenia may reflect impaired immune surveillance, rendering the host less capable of controlling aberrant inflammatory responses, particularly in the presence of exogenous or endogenous triggers such as smoking or metabolic disturbances (40, 47). Moreover, some studies have suggested that lymphopenia may be associated with reduced vagal tone and heightened sympathetic activity, an imbalance in autonomic regulation that can further destabilize atrial electrophysiology and promote AF onset (48–50). Third, elevated SIRI may also reflect inflammation-driven alterations in bone marrow hematopoiesis. Under persistent inflammatory signals, hematopoietic stem cells may be skewed toward myeloid differentiation, favoring neutrophil and monocyte lineages, while suppressing lymphopoiesis. This leads to a systemic immunological shift characterized by dominant pro-inflammatory activity and compromised adaptive immunity (51, 52). Such shifts not only contribute to higher SIRI values but may also directly influence the atrial microenvironment through the recruitment and activation of inflammatory cells, perpetuating a state of low-grade chronic inflammation that fosters atrial remodeling. Additionally, as an integrated inflammatory biomarker, SIRI is sensitive to chronic low-grade inflammation, even in the absence of overt clinical symptoms. Chronic subclinical inflammation has been increasingly recognized as a silent trigger for AF, exerting subtle yet progressive effects on atrial structural integrity and electrical conduction, thereby laying the groundwork for future AF episodes (45, 53). In summary, SIRI may play a pivotal role in the initiation and progression of AF through multiple mechanisms, including heightened activity of pro-inflammatory cells, impaired immune regulation, systemic inflammatory activation, and shifts in bone marrow hematopoiesis. Future basic and clinical studies are warranted to further elucidate the specific molecular pathways, inflammation–immune interactions, and potential therapeutic targets involved in SIRI-mediated AF pathogenesis. Such investigations may provide a theoretical foundation and practical guidance for the early identification, risk stratification, and precision intervention of AF.

Despite the valuable findings presented in this study, several limitations should be acknowledged.

First, as a retrospective cross-sectional analysis, this study is inherently unable to establish a causal relationship between SIRI and the occurrence of AF. Consequently, the identified SIRI-AF association must be interpreted strictly as correlational evidence, not causal inference–a fundamental constraint of the cross-sectional design. Prospective studies are needed to validate temporal relationships. Second, patients' medical histories and clinical data were primarily extracted from electronic medical records, which may be subject to incomplete documentation or recall bias, potentially affecting the accuracy and reliability of the results. Third, since the study population was drawn exclusively from inpatients at two tertiary hospitals, the representativeness of the sample may be limited, and selection bias cannot be ruled out, thereby restricting the generalizability of the results. Fourth, it remains challenging to fully exclude the influence of potential confounding factors, such as genetic susceptibility, dietary patterns, environmental exposures, occupational factors. Finally, as a static peripheral blood marker of inflammation, SIRI cannot dynamically capture temporal changes in inflammatory status throughout the onset and progression of AF. Its precise mechanistic role in AF pathophysiology remains unclear. Therefore, future well-designed prospective cohort studies and interventional clinical trials are warranted to clarify the temporal and causal relationship between SIRI and AF, as well as the underlying biological mechanisms.

## 5 Conclusion

Our findings indicate that elevated SIRI levels are significantly associated with an increased risk of AF in patients with COPD, with this association being particularly pronounced among smokers. These results suggest that SIRI, as a novel and potential marker of low-grade inflammation, may serve as a useful tool for assessing AF risk and guiding individualized management in COPD patients, providing a new reference for the early identification of high-risk individuals in clinical practice. Future longitudinal studies and clinical trials are needed to further validate the causal relationship and explore the potential value of SIRI as an intervention target.

## Data Availability

The data analyzed in this study is subject to the following licenses/restrictions: the datasets contain sensitive patient information and are subject to institutional and ethical restrictions. Therefore, they are not publicly available. Data may be available from the corresponding author upon reasonable request and with appropriate institutional approvals. Requests to access these datasets should be directed to zhaohongjun@wmu.edu.cn.
